# The Brief Case: hidden intruders—serendipitous discovery of an infection after appendicitis surgery

**DOI:** 10.1128/jcm.00193-25

**Published:** 2025-06-11

**Authors:** Nada Ben Halima, Brice Autier, Federica Attaianese, Maya Husain, Florence Robert-Gangneux, Estelle Sabourin, Nadia Guennouni, Eric Dannaoui, Marie-Elisabeth Bougnoux

**Affiliations:** 1 Parasitology-Mycology Unit, Clinical Microbiology Department, Hôpital Necker-Enfants Malades 246596, Paris, France; 2 Université de Rennes, CHU Rennes, INSERM, EHESP, IRSET (Institut de recherche en santé, environnement et travail) - UMR_S 1085 36684, Rennes, France; 3 Department of General Pediatrics and Infectious Diseases, Hôpital Necker-Enfants Malades 246596, Paris, France; Endeavor Health, Evanston, Illinois, USA

**Keywords:** appendicitis, tapeworm, microscopy, molecular identification, *Dibothriocephalus nihonkaiensis*, raw salmon, France

## CASE

A 14-year-old male patient from Paris presented to the emergency department with a 2-day history of abdominal pain and vomiting but no bowel transit disorders. He had no significant medical history and had not undergone any previous surgeries. On clinical examination, he was afebrile but exhibited tenderness in the right flank of his abdomen.

Laboratory investigations revealed signs of an inflammatory response, with an elevated C-reactive protein (CRP) level of 67 mg/L (normal range: 0–5 mg/L) and leukocytosis at 17.46 G/L (normal range: 3.9–10.2 G/L), with 88% neutrophils. Eosinophil levels were within the normal range, and other parameters of the complete blood count, including hemoglobin (14.1 g/dL) and mean corpuscular volume (MCV = 83.8 fL), were normal, ruling out anemia and macrocytosis. No other blood test abnormalities were noted.

An abdominal ultrasound diagnosed uncomplicated acute retrocecal appendicitis with a stercolith present. Consequently, an emergency laparoscopic appendectomy was performed.

Three days post-operation, after the patient’s bowel function resumed, he passed liquid stools containing a 3 m long tapeworm. Upon further questioning, the patient’s

mother revealed that her son experienced a previous episode of worm emission 2 years ago, which was treated with albendazole. She also reported her own history of passing “long segmented” worms in her stool 5 years earlier, similarly treated with albendazole. Neither she nor her son had experienced other symptoms, such as bowel transit disorders or abdominal pain, and no stool examination was further performed in either case to assess treatment efficacy.

The family had traveled extensively, visiting countries such as Thailand in 2017, Guadeloupe in 2018, Egypt in 2021, South Africa in 2022, and Italy on several occasions. Additionally, the mother admitted to regularly consuming raw salmon at least three times a week, as well as frequently eating in Japanese restaurants. Unfortunately, the tapeworm was discarded in the hospital facilities, making recovery for identification impossible.

**Fig 1:**
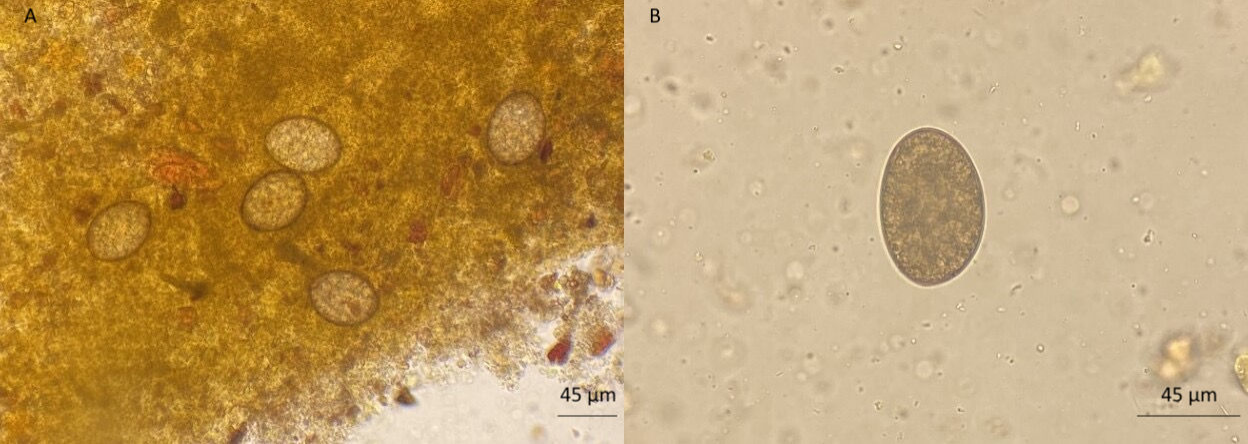
(A, B): Direct wet mount microscopy of the patient’s unstained stool specimen showing ovoid, unembryonated eggs (65 × 45 µm).

Microscopic examination of stool smears revealed numerous ovoid, unembryonated eggs measuring approximately 65 × 45 µm, with morphological features strongly indicative of a diphyllobothriid cestode (Fig. 1).

Results obtained with the multiplex PCR Allplex GI-Parasite and Allplex GI-Helminth assays (Seegene) were negative, while the NovoDiag Stool Parasite multiplex PCR detected the presence of *Dibothriocephalus nihonkaiensis/latus* DNA in the stool sample. The final identification was confirmed based on mitochondrial cytochrome c oxidase subunit I (*cox1*) gene sequencing using Sanger method and the JB6/JB5R primers, as previously described (1). The obtained 606 bp sequence allowed us to identify the causative agent as *Dibothriocephalus nihonkaiensis* (GenBank accession number PV153717), with >99% identity to reference sequences from Genbank and 100% coverage. The sequencing analysis was performed by the laboratory of Parasitology of the Rennes University Hospital (France), per routine testing.

Histopathological examination of appendectomy specimens revealed acute appendicitis. The pattern of inflammation was predominantly suppurative, with evidence of a transmural inflammatory process and mild periappendicular peritonitis, likely caused by the stercolith. No eggs were observed in the appendiceal tissue.

The patient was definitively diagnosed with diphyllobothriasis caused by *Dibothriocephalus nihonkaiensis*. He was treated with a single dose of praziquantel at 10 mg/kg while hospitalized. The tapeworm was considered eliminated, as no further worm or proglottid release was observed post-treatment. The patient reported no residual abdominal pain. The control stool examination was negative months after treatment.

## DISCUSSION

Human diphyllobothriasis is an intestinal parasitic disease caused by diphyllobothriid tapeworms, typically contracted by consuming raw or undercooked fresh or marine water fish (2). It represents the most common fish-borne cestodiasis, with an estimated 20 million people infected worldwide (3). Human-infecting diphyllobothriid cestodes are classified into three genera: *Dibothriocephalus*, *Adenocephalus*, and *Diphyllobothrium*, the latter being the least common in humans. *Dibothriocephalus* is the most frequently reported, with *D. latus* as the most prevalent, originally found in temperate areas of the Holarctic, followed by *D. nihonkaiensis* (4, 5).

Initially described in Japan, *D. nihonkaiensis* uses anadromous Pacific salmonids (*Oncorhynchus masou, O. keta, O. gorbuscha,* and *O. nerka*) as predator fish of second intermediate hosts, usually small fish. It is the most common causative agent of diphyllobothriasis in the North Pacific coast of Asia (Russia, China, Japan, and South Korea) and North America (Canada and USA), but imported cases have been reported in Europe and New Zealand (5).

In France, most cases are due to *D. latus*, with only 11 cases of *D. nihonkaiensis* reported between 2005 and 2021, likely due to increasing consumption of raw salmon, as commonly consumed in Japanese restaurants (6). The first French case of *D. nihonkaiensis* was reported in Nice in 2005 (2), followed by a second case in Paris in 2007 (7). From 2016 to 2018, seven cases were identified consecutively in Rennes (1), and in 2019, another case was reported in Strasbourg (8). The 11th case was diagnosed in Île-de-France in 2020 (6). Epidemiological investigations suggested that these infections had been likely acquired in France due to the consumption of imported sushi, given the absence of a travel history to areas of endemicity.

Although most cases of diphyllobothriasis are asymptomatic during weeks after consumption of the infected meal, abdominal discomfort, diarrhea, vomiting, weakness, and weight loss may occur (5). Thus, except when proglottids are released with feces, the disease is rarely suspected. Rare complications, such as intestinal obstruction or erratic migration—occasionally obstructing lumina and causing cholangitis, appendicitis, or cholecystitis—can occur, mainly in cases of massive infestation (9). Prolonged or heavy diphyllobothriasis can exceptionally lead to megaloblastic anemia due to vitamin B12 deficiency, resulting from dissociation of vitamin B12 from the host intrinsic factor complex in the gut. The tapeworm absorbs vitamin B12 100 times faster than the human gut (3). Nevertheless, megaloblastic anemia due to *D. nihonkaiensis* infection has never been documented among the French cases, nor in those reported in the international literature (6).

In this case, diphyllobothriasis was an incidental finding, along with acute appendicitis, attributed to appendicular lumen obstruction by a stercolith, visualized on ultrasound and confirmed pathologically. Nevertheless, the presence of the worm in the appendiceal lumen could also contribute to appendicitis, as reported in a case of *D. latus* infection presenting with chronic right lower abdominal pain mimicking subacute appendicitis. In this previously published case, colonoscopic images revealed the tapeworm lodged in the appendiceal orifice (10).

Although diphyllobothriasis is not life-threatening, accurate diagnosis and species identification are essential, not because treatments differ by species—they are generally treated similarly—but to update epidemiological data, monitor species geographic distribution, and guide infection control strategies (9).

Diagnosis relies on detecting proglottids or eggs in stool specimens, which are characteristic of diphyllobothriid cestodes but are insufficient for identification at species or even genus level (9). Measuring eggs is important to avoid misidentification of morphologically similar trematode eggs. Identification to the species level requires molecular methods, especially sequence analysis of the mitochondrial cytochrome c oxidase subunit I gene (cox1) (5).

Based on morphological criteria, the eggs observed in our patient’s stool were first identified as Diphyllobothriidae. Molecular analysis was necessary to refine identification.

Our routine Seegene multiplex PCR assays, Allplex GI-Parasite and Allplex GI-Helminth, returned negative results. These assays enable the detection of six protozoa and eight helminths and microsporidia, respectively, in stool specimens. The Allplex GI-Parasite Assay targets *Giardia duodenalis*, *Cryptosporidium* spp., *Entamoeba histolytica*, *Dientamoeba fragilis*, *Blastocystis* spp., and *Cyclospora* spp. The Allplex GI-Helminth Assay detects *Ancylostoma* spp., *Ascaris* spp., *Enterobius vermicularis*, *Hymenolepis* spp., *Necator americanus*, *Strongyloides* spp., *Taenia* spp., and *Trichuris trichiura*, alongside microsporidia (*Enterocytozoon* spp. and *Encephalitozoon* spp.). This explains the negative results, as *Dibothriocephalus* is not included in the assay panels. Furthermore, there is no information regarding potential cross-reactivity within the Diphyllobothriidae family.

Both assays exhibit variable sensitivity and specificity depending on the targeted parasite (11, 12). The diagnostic performance of the Allplex GI-Helminth Assay remains incompletely characterized, with limited validation data available. A recent study by Coppens et al. reported comparable findings, although based on a small number of positive samples (13). Further studies are required to refine its diagnostic accuracy.

The Novodiag Stool Parasites Assay (Mobidiag, Espoo, Finland), a novel automated approach combining real-time PCR and microarray technologies, enables high-plex detection of 26 distinct targets, encompassing protozoans, helminths, and two microsporidian genera, from unpreserved stools. While it offers rapid results within 90 min, the assay provides only qualitative results, limiting its relevance for therapeutic follow-up. Recent studies have demonstrated higher sensitivity of the Novodiag Stool Parasites Assay compared to routine procedures for *Blastocystis* spp., *D. fragilis*, *Schistosoma* spp., and *Enterobius vermicularis*, though false positives were noted for *Blastocystis* spp., *G. duodenalis*, and *Trichuris* spp. (14).

Today, the performance of available multiplex PCR assays at detecting *D. nihonkaiensis*, including comparisons between microscopy and molecular methods, is understudied. *Dibothriocephalus* is not currently included on commercially available multiplex molecular panels for rapid identification from stool specimens.

The final identification of *D. nihonkaiensis* was performed using molecular methods based on Cox1 gene sequencing.

This case highlights the importance of classical parasitology techniques, which were crucial in the initial detection of the infection. These methods remain the gold standard for diagnosing Diphyllobothriidae at the family level by detecting eggs or proglottids in stool specimens. This case also illustrates how routine multiplex PCR alone would have missed the diagnosis. Although multiplex PCR assays offer the advantage of a syndromic testing approach and rapid screening, they are limited by their ability to identify only a defined number of targets. Thus, physicians and primary care providers should be aware of possible diagnostic gaps resulting from the use of multiplex assays and refer patients to specialized laboratories when a parasitic infection is strongly suspected, as in the present case, to ensure accurate identification using appropriate molecular tools (8). Interestingly, in this case, the observation of the worm prompted further investigations, which ultimately led to the accurate diagnosis, as detailed.

Diphyllobothriasis is easily cured with a single oral dose of praziquantel (5 to 10 mg/kg dose in children and adults) as recommended by the Centers for Disease Control and Prevention (CDC). A single dose of niclosamide (2 g for adults, 50 mg/kg up to a maximum of 2 g for children) is an alternative; however, this treatment is no longer available in France (15). Treatment efficacy should be verified 3 to 4 months post-treatment via microscopic stool examination (9). Importantly, infection prevention is based on cooking (≥55°C) or freezing (-8°C for 12 h or -10°C for 6 h) raw fish (6). In our case, the previous treatment with albendazole administered to the mother and her son was not optimal, raising the possibility of persistent or recurrent infection.

In conclusion, this case exemplifies the emergence of *D. nihonkaiensis* infections in non-endemic regions like France, likely due to the consumption of raw fish imported from endemic areas. It highlights the need for physicians and primary care providers to consider diphyllobothriasis as a possible etiology of intestinal disorders in patients with a history of raw fish consumption. Enhanced awareness, combined with the appropriate use of molecular and classical parasitological techniques, is essential for accurate diagnosis and effective public health management.

## SELF-ASSESSMENT QUESTIONS

1. In the life cycle of *Dibothriocephalus nihonkaiensis*, what is the role of Pacific salmon?
  - Definitive host
  - First intermediate host
  - Paratenic host
  - Mechanical vector
2. Which of the following laboratory methods is most specific for identifying *Dibothriocephalus nihonkaiensis* infection?
  - Detection of characteristic eggs or proglottids in stool specimens using microscopy
  - Serological testing for *Dibothriocephalus* specific antibodies
  - Routine multiplex PCR assays
  - Mitochondrial Cox1 gene sequencing
3. Which of the following is a recommended strategy to prevent *Dibothriocephalus nihonkaiensis* infection?
  - Salting salmon for at least 48 h
  - Refrigerating salmon at +4°C for 2 days
  - Cooking salmon to an internal temperature of 55°C or above
  - Marinating salmon in vinegar or lemon juice for 30 min

## ANSWERS TO SELF-ASSESSMENT QUESTIONS

1. In the life cycle of *Dibothriocephalus nihonkaiensis*, what is the role of Pacific salmon?
  - Definitive host
  - First intermediate host
  - Paratenic host
  - Mechanical vector

Answer: c. Paratenic host.

The life cycle of *D. nihonkaiensis* is similar to that of other Diphyllobothriid tapeworms. It begins when eggs are excreted by the definitive host (e.g., humans) in feces, which are released into fresh or marine water, where they develop into first larvae (coracidium). These coracidia are ingested by the first intermediate crustacean host, typically copepods, where they evolve into procercoid larvae. The copepods are consumed by the second intermediate host, usually small fish. In the second intermediate host, procercoid larvae develop into plerocercoids. The second intermediate host is ingested in this case by Pacific salmonids, which serve as paratenic hosts. In such hosts, there is no further development of the larvae, but they remain infective to the definitive host. Humans become infected by consuming raw or undercooked fresh or marine water fish containing these plerocercoids. Mature tapeworms develop once the plerocercoids attach, usually in the ileum. They grow into folded loops in the small intestine, producing their first eggs 2 to 6 weeks later. Adults produce millions of eggs per day expelled with host feces, beginning the life cycle anew.

- Which of the following laboratory methods is most specific for identifying *Dibothriocephalus nihonkaiensis* infection?
  - Detection of characteristic eggs or proglottids in stool specimens using microscopy
  - Serological testing for *Dibothriocephalus* specific antibodies
  - Routine multiplex PCR assays
  - Mitochondrial Cox1 gene sequencing

Answer: d. Mitochondrial Cox1 gene sequencing

Microscopy can provide initial orientation towards the Diphyllobothriidae family by detecting characteristic eggs or proglottids in stool specimens but cannot differentiate species within the genus. Serological testing is not used for tapeworms. Routine multiplex PCR assays generally target more common gastrointestinal pathogens, which means Diphyllobothriid tapeworms may not be included, limiting their utility. Molecular testing such as sequencing of the mitochondrial Cox1 gene enables species confirmation and epidemiological monitoring.

- Which of the following is a recommended strategy to prevent *Dibothriocephalus nihonkaiensis* infection?
  - Salting salmon for at least 48 h
  - Refrigerating salmon at +4°C for 2 days
  - Cooking salmon to an internal temperature of 55°C or above
  - Marinating salmon in vinegar or lemon juice for 30 min

Answer: c. Cooking salmon to an internal temperature of 55°C or above effectively kills the plerocercoid larvae responsible for infection. Freezing salmon at -8°C for 12 h or -10°C for 6 h can also inactivate the larvae. However, marinating or salting fish is insufficient to ensure safety. European regulations for food workers recommend freezing raw fish at temperatures lower than -20°C for at least 24 h to prevent potential contamination with fish parasites, as outlined in Regulation N° 853/2004 of the European Parliament.

**TAKE-HOME POINTS**

- The emergence of diphyllobothriasis cases caused by *D. nihonkaiensis* in non-endemic countries, such as France is due to an increase in the export of salmon from the North Pacific coast and the growing fashion of consuming it raw or undercooked.
- *D. nihonkaiensis* tapeworms are rare but likely underestimated given the lack of systematic molecular diagnosis in the event of *D. latus* based on microscopy.
- Microscopic identification of eggs or proglottids in stool is the basis of family level diagnosis. However, based on routine microscopy, diphyllobothriid cestodes are often reported as *D. latus*, in the absence of molecular confirmation. Accurate species-level identification requires molecular methods and is mainly useful for epidemiological knowledge.
- Biologists should be aware of molecular methods, understanding both their performance and limitations in order to use the appropriate tools for diagnosis.
- Public education by public health programs and food safety measures is essential to reduce the risk of diphyllobothriid cestode infection as well as other parasites, e.g., anisakids.
